# Fundoplication in Patients with Esophageal Atresia: Patient Selection, Indications, and Outcomes

**DOI:** 10.3389/fped.2017.00109

**Published:** 2017-05-15

**Authors:** Risto J. Rintala

**Affiliations:** ^1^Children’s Hospital, Helsinki University Central Hospital, Helsinki, Finland

**Keywords:** esophageal atresia, fundoplication, anti-reflux surgery, gastroesophageal reflux, anastomotic stricture, acute life-threatening events, long-gap atresia

## Abstract

Patients with esophageal atresia (EA) suffer from abnormal and permanent esophageal intrinsic and extrinsic innervation that affects severely esophageal motility. The repair of EA also results in esophageal shortening that affects distal esophageal sphincter mechanism. Consequently, gastroesophageal reflux (GER) is common in these patients, overall approximately half of them suffer from symptomatic reflux. GER in EA patients often resists medical therapy and anti-reflux surgery in the form of fundoplication is required. In patients with pure and long gap EA, the barrier mechanisms against reflux are even more damaged, therefore, most of these patients undergo fundoplication during first year of life. Other indications for anti-reflux surgery include recalcitrant anastomotic stenoses and apparent life-threatening episodes. In short term, fundoplication alleviates symptoms in most patients but recurrences are common occurring in at least one third of the patients. Patients with fundoplication wrap failure often require redo surgery, which may be complicated and associated with significant morbidity. A safe option in a subset of patients with failed anti-reflux surgery appears to be long-term medical treatment with proton pump inhibitors.

## Introduction

The esophagus is not normal following repair of an esophageal atresia (EA). The motility of the esophagus is permanently altered, and the esophagus is usually shorter than normal (1–3). The tension and abnormal perfusion at the anastomotic site commonly cause stricture formation that requires anastomotic dilatations. Pathological gastroesophageal reflux (GER) that is caused by shortening of the esophagus and abnormal clearance of esophageal contents due to abnormal motility affects up to two thirds of patients with EA (1, 4). Some EA patients experience acute life-threatening events (ALTE) that may be associated with proximal extension of GER and also with tracheomalacia that commonly accompanies EA. Recurrent respiratory disease has been attributed to GER but evidence supporting this is not convincing. Medical therapy, today mainly by proton pump inhibitors (PPI), is always the first-line approach for these patients but a significant percentage ultimately undergoes surgery in the form of fundoplication. Most pediatric surgeons agree that patients with pure or long-gap EA very often require fundoplication to overcome severe GER and anastomotic strictures associated with the significant shortening of the esophagus. In the literature, the overall rate of fundoplication in patients with EA ranges between 10 and 45% (1, 4, 5).

## Indications for Fundoplication in EA

### Gastroesophageal Reflux Disease (GERD)

The abnormal esophageal anatomy after repair of EA plays a significant role in the etiology of GERD. The esophageal repair often causes esophageal shortening that may displace the gastroesophageal junction upward causing an obtuse angle of His. This is especially true in patients with long-gap atresia and significant anastomotic tension (6).

The esophageal peristalsis that is responsible for esophageal clearance is damaged in patients with EA (2, 3). The abnormal and ineffective peristalsis does not improve by age as most adult patients with repaired EA still show highly abnormal and decreased motility in manometric studies (5). The cause of poor motility is probably multifactorial. The arrangement of muscular layers may be abnormal in EA (1). Both extrinsic and intrinsic innervation of the esophageal wall is congenitally deficient (7, 8), and there is additional damage that is caused by the extensive dissection required for the making of esophago-esophageal anastomosis (9).

Symptomatic GER is very common in infants with EA, the incidence ranges between 25 and 70% (1). Moreover, unlike GER that is not associated with anatomical defects, the proportion of significant EA-associated GER tends to increase over time (10). GER in infants with EA does not respond well to standard methods of management such as thickening of milk and postural treatment. Medical treatment may also be unsuccessful although most pediatric surgeons routinely treat their EA patients with long-term anti-acid medication, today usually with PPI.

Of patients who suffer from significant GERD 30–64% undergo fundoplication. Most patients require fundoplication before the age of 1 year. There are no generally accepted indications for fundoplication in EA patients who suffer from significant GER. The usual causes leading to operation are failure of medical treatment to control symptoms, failure to thrive, and GER-related refractory anastomotic stenosis.

### Anastomotic Stricture

Anastomotic strictures requiring dilatation occur in 30–60% of EA patients (4, 5, 11). Most strictures respond well to anastomotic dilatations but the choice of the timing of dilatations and the number of dilatations remain arbitrary. Most pediatric surgeons dilate only symptomatic patients. A small proportion of patients who suffer from recalcitrant strictures are commonly considered to have significant GER that contributes to refractory stenosis formation. Anti-reflux surgery has been suggested to be curative in most of these patients (12, 13). However, there are no scientifically based definitions for recalcitrant strictures or for the timing of surgery. The surgeon’s judgment based on personal or institutional experience dictates the timing of surgery. Moreover, the efficacy of fundoplication in the management of anastomotic strictures remains scientifically unproven. This is especially true today in the era of PPI’s that are more or less routinely used in patients with EA (14).

### Pure/Long-Gap EA

There is a lack of generally accepted definition for long-gap EA. Some surgeons consider only pure (type A) or type B atresia with proximal fistula as long-gap atresia, some include also “long-gap” type C (with distal fistula) atresia. There are also no uniformly accepted methods to measure the gap between the esophageal ends. It has been clearly shown that long-gap predisposes to symptomatic GERD and anastomotic strictures (4, 6, 15), mainly because of considerable tension in the anastomosis. The recent esophageal lengthening techniques are associated with GERD, and fundoplication is required in most patients (16). Anti-reflux surgery is considered as a routine and predictable step in the management of long-gap EA patients by some surgeons (6, 16, 17), others perform fundoplication only in patients with severe symptoms and abnormal GERD tests (15).

### Acute Life-Threatening Events

Acute life-threatening events in the form of cyanotic or dying spells occur in some patients with EA. The actual incidence is not very well documented but operative treatment is required in 5–12% of patients (15, 18). The pathophysiology of ALTE in patients with EA is not fully understood. Many of these patients have significant tracheomalacia, which is commonly associated with GERD. In the literature, ALTE is considered as an absolute indication for surgical treatment (19, 20). There is no consensus concerning the optimal management of ALTE. In the presence of tracheomalacia, some surgeons perform primary aortopexy that may be followed by fundoplication (19, 20), some favor simultaneous aortopexy and fundoplication (18). Some patients may be treated by fundoplication alone if the etiology of ALTE is considered to be mainly GERD (21).

### Severe Respiratory Disease

Up to 74% of patients with repaired EA suffer from chronic or recurrent respiratory symptoms (22). Pulmonary lung function test has revealed that 70–90% of EA patients have detectable ventilatory impairment. The defect may be restrictive or obstructive or both (22). Moreover, a significant proportion of patients have abnormal airway reactivity suggesting susceptibility to asthma. It appears, however, that these symptoms are not related to GER (23). In addition, fundoplication has not been shown to protect from respiratory symptoms or ventilator defects (24). Anti-reflux surgery probably has no role in the management of respiratory disease in EA patients.

## Preoperative Work-Up

In most cases, the decision to perform fundoplication in patients with EA is based on clinical symptoms and findings. Diagnostic tests are not always helpful but may support decision-making in selected cases. Esophagogastroduodenoscopy is helpful in detecting inflammatory changes in the esophagus of patients with symptomatic GER. Detection of chronic inflammation in symptomatic EA patients is considered to support surgical therapy. Endoscopy is also useful to assess the severity of anastomotic stricture and its response to dilatations. Esophageal pH-metry and impedance pH-metry may be useful adjuncts in surgical decision-making; high reflux indices support surgical therapy in a symptomatic patient. Esophageal manometry is usually not applicable and is anyway almost always pathological in EA patients (22). Gastric emptying studies are often abnormal in EA patients and not very useful in clinical practice.

## What Type of Anti-Reflux Surgery for EA Patients?

The selection of the type of anti-reflux surgery in patients with EA has been a matter of debate between pediatric surgeons. Partial wraps such as Thal (anterior wrap) or Toupet (posterior wrap) operation may be associated with less adverse effect, but a higher failure rate (25, 26). On the other hand, complete fundoplication such as Nissen operation may result in more dysphagia, retching, and gas–bloat (27). This is, however, not supported by solid scientific evidence, and some studies have not found any differences between complete and partial wraps (28). There is absolutely no consensus as to whether partial or complete fundoplication should be used in patients with EA. There is even less valid scientific evidence to support superiority of either approach in this patient population. Anti-reflux surgery may be performed laparoscopically with similar success rate than in open surgery, whether with partial or complete hiatal wrap (29).

Practically, all patients with EA have abnormal esophageal motility (2) that makes them a special group compared to otherwise healthy patients requiring fundoplication. They have more often esophagitis and higher rate of strictures, and they have commonly delayed gastric emptying. The motility problems predispose EA patients to postoperative dysphagia and ultimately to wrap failure. Some patients may not be able to generate enough propulsion to overcome the increased resistance at the esophagogastric junction created by the fundoplication and may develop respiratory tract problems caused by regurgitation of esophageal contents (30). Postoperative dysphagia is typical for laparoscopic anti-reflux surgery occurring in one third of the patients (29), but it usually disappears within a couple of months.

Some surgeons prefer to use esophageal lengthening procedures in association with anti-reflux surgery (31). The most popular approach is the Collis–Nissen procedure where the esophagus is lengthened by stapling the esophagogastric junction longitudinally. This operation is mainly used in redo surgery. The main problem with this procedure is that it leaves acid secreting mucosa in the chest that may result in the development of chronic esophagitis and Barrett’s esophagus.

## Outcomes of Fundoplication in EA

Typically, in most EA patients who have undergone anti-reflux surgery the symptoms are initially alleviated (17, 32, 33). Unfortunately, the positive effect of fundoplication is transient in a significant proportion of patients. Partial wrap may be associated with fewer symptoms at least after short-term follow-up (25), but the scientific basis remains vague.

The wrap failure rates range between 20 and 45% (17, 32–35). This is significantly higher rate than in those who undergo fundoplication without any underlying anatomic defect (33). The wrap failure is usually detected 1.5–2.5 years following the primary fundoplication (17). The failure rate appears to be similar for both complete and partial wraps or open and laparoscopic approaches. The main problem in the literature is that the wrap failure is poorly defined. Most studies define failure as a need for reoperation but the actual reasons for reoperation are not fully described. The length of follow-up, symptoms, investigations, and findings leading to a decision to redo the fundoplication are inconsistently characterized in the literature. The main reason for these problems is that all studies on the fate of fundoplication in EA patients are retrospective. As wrap failure is usually defined as need to redo the fundoplication, it is likely that the actual failure rate is much higher as patients with milder symptoms are most likely managed conservatively. Moreover, if all patients would undergo regular and long-term endoscopic follow-up, the anatomical failure rate (wrap failure and thoracic dislocation of the wrap) would be significantly higher than reported.

The causes of wrap failure are likely to be the same anatomical and physiological abnormalities that have caused GERD in these patients. The short length and poor propulsive activity of the repaired esophagus interfere very likely with the persistence of the fundoplication (34). The stomach may also be smaller than normally, especially in patients who originally have had a pure type A EA, which may influence performing of a reliable fundoplication. Delayed gastric emptying is a common and persisting finding in EA patients and may contribute to high incidence of wrap failures (36).

## What are the Options When Fundoplication has Failed?

The high incidence of wrap failure following primary fundoplication in patients with EA raises the question: what to do next? For pediatric surgeons, the natural response is to do a redo operation to correct the failed wrap (17, 34, 37). Diagnostic work-up is required in patients with symptoms of wrap failure. The typical tests are contrast X-ray studies, upper gastrointestinal endoscopy, and pH-metry. The typical findings at imaging and endoscopy are partial or complete unwrapping of the fundoplication or dislocation of the wrap partially or completely into the chest. pH-metry usually shows a high reflux index compared with previous postoperative measurements.

Reoperation following failed wrap is significantly more demanding than the primary fundoplication. There is always major scarring and adherence of the stomach and wrap area to the spleen, liver, and diaphragm. The operative times are longer, and blood loss and postoperative complication rates are increased (35, 38).

The literature offers very little data on the outcomes of re do fundoplication. Redo fundoplication has been reported to be successful in 70–80% of cases overall (35, 38); however, the failure rate may be higher than following the primary operation, especially in patients with EA (34). This is not unexpected because the factors that have caused the failure of the primary wrap are still present. It appears to be imperative to put effort in patient selection for redo fundoplication.

An alternative to repeat fundoplication may be maintenance therapy with PPI’s. Although prophylactic PPI therapy does not reduce the incidence of anastomotic stenosis in infants who have undergone repair of EA (39, 40), PPI’s can induce long-term remission of erosive esophagitis (41). Marked improvement has been noted in symptoms of GERD and severity of esophagitis in patients who have received PPI maintenance therapy after failed fundoplication (42). Long-term maintenance therapy has also been shown to be safe with few adverse effects (43).

Severely symptomatic patients who have undergone one or several redo fundoplications and who often do not tolerate oral feeding or feedings through gastrostomy are a problematic group in EA patients. These patients often suffer from associated malformations or syndromes and have often undergone multiple revisional operations (44). Feeding jejunostomy may decrease GER-related symptoms and provides a route for enteral feeding at least temporarily for this unfortunate group. Another option is esophagogastric disconnection that has been used as rescue therapy following failed fundoplications (45). Esophagogastric disconnection provides a reliable route for gastrostomy feedings and may eliminate GER and its consequences completely.

## Conclusion

Fundoplication is frequently required in EA patients, however, the indications for fundoplication are not scientifically delineated. Partial wraps may be associated with better functional outcome but, again, the scientific basis for the statement is vague. This clinical equipoise calls for multicenter randomized controlled studies to evaluate partial and complete wraps in EA patient population. After fundoplication most patients have excellent relief of their symptoms. However, wrap failure is much more common than in patients without EA and is not related to the type of fundoplication. Many patients with wrap failure require redo surgery but long-term PPI therapy deserves to be considered before subsequent surgical intervention.

## Author Contributions

RR reviewed the literature and wrote the manuscript.

## Conflict of Interest Statement

The author declares that the research was conducted in the absence of any commercial or financial relationships that could be construed as a potential conflict of interest.
